# Topological Complexity Determines the Frequency of Branching Variations in the Celiac Trunk–Superior Mesenteric Artery System

**DOI:** 10.1002/jhbp.70120

**Published:** 2026-04-23

**Authors:** Satoru Muro, Akimoto Nimura, Keiichi Akita

**Affiliations:** ^1^ Department of Clinical Anatomy Graduate School of Medical and Dental Sciences, Institute of Science Tokyo Tokyo Japan; ^2^ Department of Functional Joint Anatomy Biomedical Engineering Laboratory, Institute of Industry Incubation, Institute of Science Tokyo Tokyo Japan

**Keywords:** anatomical variation, arterial branching, celiac trunk, hepatobiliary surgery, superior mesenteric artery

## Abstract

**Background:**

Variations in the anatomical branching patterns of the celiac trunk and superior mesenteric artery (SMA) directly affect vascular identification, surgical planning, and risk of intraoperative vascular injury. However, despite the extensive classification of these variations, the principles governing the occurrence of certain branching patterns remain unclear.

**Methods:**

We analyzed 28 branching subtypes of the celiac trunk–SMA system described by Adachi. Each branching pattern was quantified relative to the standard configuration by using Node‐Shift (relative displacement of branching nodes), Edge‐Gain (number of additional branches), and Edge‐Loss (number of missing branches). The relationships between these topological parameters and branching frequency were examined using linear, log‐linear, and exponential regression models.

**Results:**

Branching frequency consistently decreased as topological complexity increased. In subtypes without branch loss, the branching frequency showed a strong exponential decline with increasing Node‐Shift and Edge‐Gain (*R* ^2^ = 0.979). When all subtypes were included, the exponential model maintained a similarly high explanatory power (*R* ^2^ = 0.979), whereas the linear and log‐linear models showed a limited fit. Branch loss was exclusively associated with very‐low‐frequency patterns.

**Conclusions:**

Arterial branching variations do not arise in a purely random manner and are consistent with the probabilistic constraints of developmental and anatomical pattern formation.

## Graphical abstract

Muro and colleagues reanalyzed Adachi's 28 celiac trunk–superior mesenteric artery branching patterns using three parameters: Node‐Shift, Edge‐Gain, and Edge‐Loss. Branching frequency decreased exponentially as topological complexity increased, indicating a probabilistic structure shaped by developmental and anatomical constraints rather than chance, with implications for precision anatomy in hepatobiliary–pancreatic surgery.

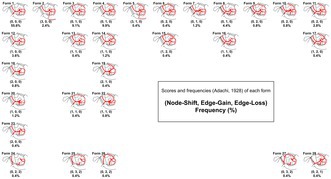

## 1 Introduction

Variations in the branching patterns of the celiac trunk and superior mesenteric artery (SMA) directly influence the identification of major vessels and the safety of vascular management during hepatobiliary–pancreatic surgery. Accordingly, the morphological diversity of these arterial branches has attracted the attention of anatomists and surgeons [1, 2]. Anatomical studies have classified the branching patterns of the celiac trunk–SMA system on the basis of large case series and documented their occurrence frequency in detail [3, 4, 5, 6]. These data form an essential foundation for understanding the diversity of human vascular anatomy.

A re‐examination of the course and distribution of abdominal vessels in relation to membranous structures, such as the peritoneum, mesentery, and fusion fasciae, suggests that the spatial constraints imposed by these membranes influence vascular trajectories [7, 8, 9]. However, within these constraints, the extent to which specific branching patterns are determined or whether they arise in an essentially random manner remains unclear. In particular, no quantitative or systematic theoretical framework has been established to explain the differences in the occurrence frequency of branching patterns.

Arterial branching patterns are defined more by branch connectivity (i.e., which arteries connect to which points) than by geometric features, such as vessel length or branching angles. In line with this framework, classical classifications and contemporary descriptions of anatomical variations have characterized branching types primarily on the basis of topological structures [1, 2, 10]. More recently, we reported that the “topological distance” between aortic arch branching patterns shows an exponential relationship with their occurrence frequency [11]. Therefore, the variations in arterial branching patterns are not governed by complete randomness but instead follow inherent regularities embedded within the connectivity structure itself. This observation motivated the hypothesis that a similar mathematical principle might also apply to the branching variations of the celiac trunk and SMA.

In this study, we aimed to elucidate the underlying regularities governing the occurrence of branching variations in the celiac trunk–SMA system. We developed novel scoring parameters to quantitatively characterize branching patterns from a topological perspective and formally defined each branching type by using these indices. Thereafter, we analyzed the relationship between these topological scores and reported occurrence frequencies by using mathematical modeling. On the basis of this approach, we propose a new explanatory framework for understanding how branching variations in the celiac trunk and SMA arise.

## 2 Methods

### 2.1 Classification System

**FIGURE 1:**
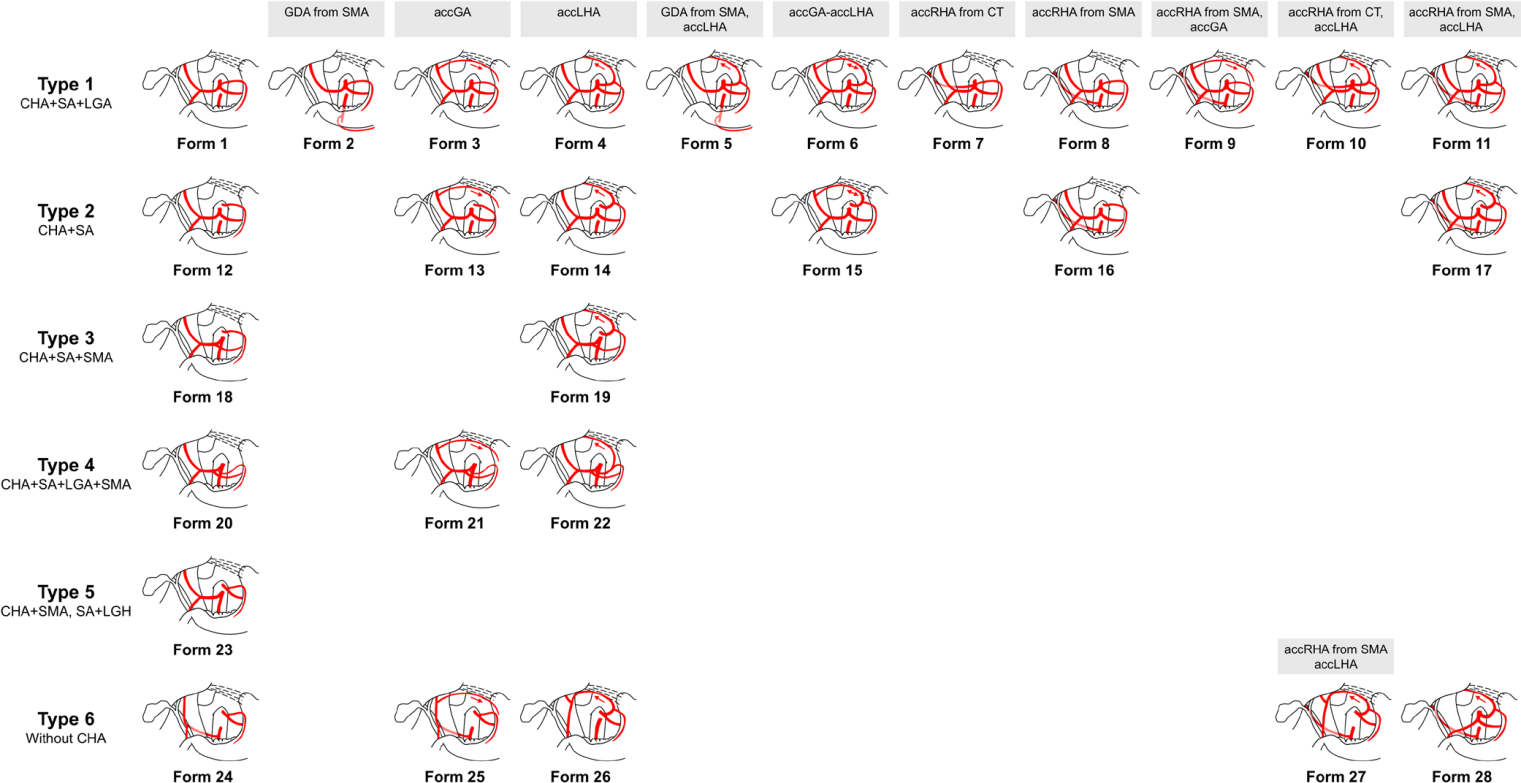
Adachi classification of the celiac trunk–superior mesenteric artery (SMA) arterial system. Schematic overview of the classical Adachi classification (1928) of the branching patterns of the celiac trunk and SMA. The arterial configuration was classified into six major types according to the relationships between the common hepatic artery (CHA), splenic artery (SA), left gastric artery (LGA), and SMA. Each type was further subdivided into 28 forms on the basis of variations in the distal branches, including the gastroduodenal artery (GDA), accessory gastric, and accessory hepatic arteries. Type 1 (Form 1) was regarded as the standard arterial configuration and used as the topological reference in the present study.

To define the branching patterns of the celiac trunk and the SMA, we adopted the classical classification proposed by Adachi [1] (Figure 1). In this system, the overall branching configuration is first categorized into six major types on the basis of the relationships between the principal arteries, namely, the common hepatic artery (CHA), splenic artery (SA), left gastric artery (LGA), and SMA. Each type is then further subdivided into 28 subtypes (forms) according to differences in the more distal branches, including the gastroduodenal, accessory gastric, and accessory hepatic arteries. Type I (Form 1) is regarded as the standard configuration.

The Adachi classification represents the most comprehensive and systematically organized description of branching patterns in the celiac trunk–SMA system. Therefore, we used this classification to provide a clear and consistent structural framework for the topological analysis of the arterial branching patterns included in this study.

### 2.2 Frequency Data

Data on the occurrence frequency of branching patterns were obtained from the large‐scale anatomical study by Adachi [1], which examined 252 adult cadavers. In that study, the branching configurations of the celiac trunk–SMA system were identified by gross anatomical dissection and classified according to the aforementioned scheme, with the number of cases and relative frequencies documented for all 28 subtypes.

In this study, these published aggregate data were used as primary frequency data and were reanalyzed to evaluate the mathematical relationship between the occurrence frequency of each subtype (form) and the topological parameters defined below.

### 2.3 Definition of Topological Parameters

**FIGURE 2:**
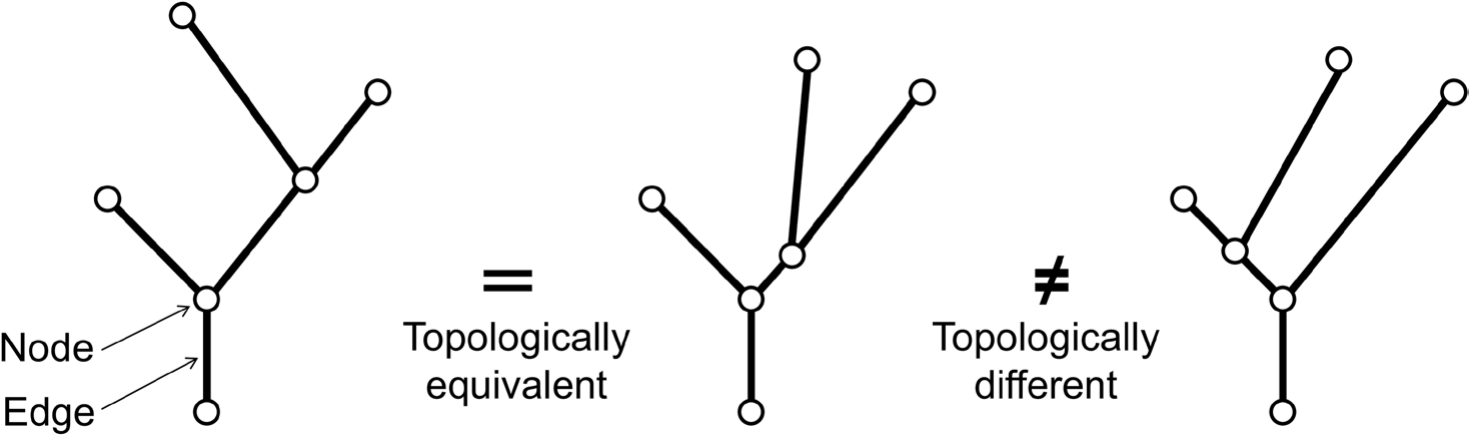
Topological equivalence in arterial branching patterns. Conceptual illustration of the topological equivalence and differences in arterial branching patterns. Arterial trees are networks comprising nodes (branching points) and edges (arterial segments). Variations in the spatial course or geometry of the arteries do not alter the topology as long as connectivity is preserved. Topological differences arise only when the branching connectivity is altered, such as by the addition, loss, or relocation of arterial branches.

Arterial branching structures can be represented as networks consisting of nodes (branching points) and edges (vascular segments). Even if the spatial positions of the nodes or the lengths and angles of the edges change, the topology of the structure remains unchanged as long as the connectivity between the nodes and edges is preserved. Topological differences arise only when these relationships are altered (Figure 2). Therefore, this concept is well‐suited for capturing the essential structural characteristics of vascular branching patterns.

From a developmental perspective, vascular branching is thought to arise through the elongation and remodeling of an initial vascular plexus, involving processes such as sprouting, regression, branching, and fusion [12, 13]. The addition or loss of branches during this process reflects changes in connectivity rather than simple positional shifts, and these events can be fundamentally understood as topological events. When the most frequently observed branching pattern is defined as the standard configuration, patterns with a greater number of branch additions or losses can be regarded as having larger topological deviations from the standard.

On the basis of this concept, we defined three parameters to quantify the topological complexity of the branching patterns. The Node‐Shift Score follows the same concept as that used in our previous study [11], whereas the Edge‐Gain and Edge‐Loss Scores were derived by simplifying and redefining the Edge‐Loss‐Change Score from that study to better suit the celiac trunk–SMA system, which exhibits limited segmental repetition.

In this framework, branching nodes were defined as the origins of the principal arteries in the celiac trunk–SMA system. These included the origins of the LGA, CHA, SA, and SMA, as well as the celiac trunk itself, which served as the reference branching point. These nodes constitute a predefined arterial branching graph used as the reference structure for topological comparison.

**FIGURE 3:**
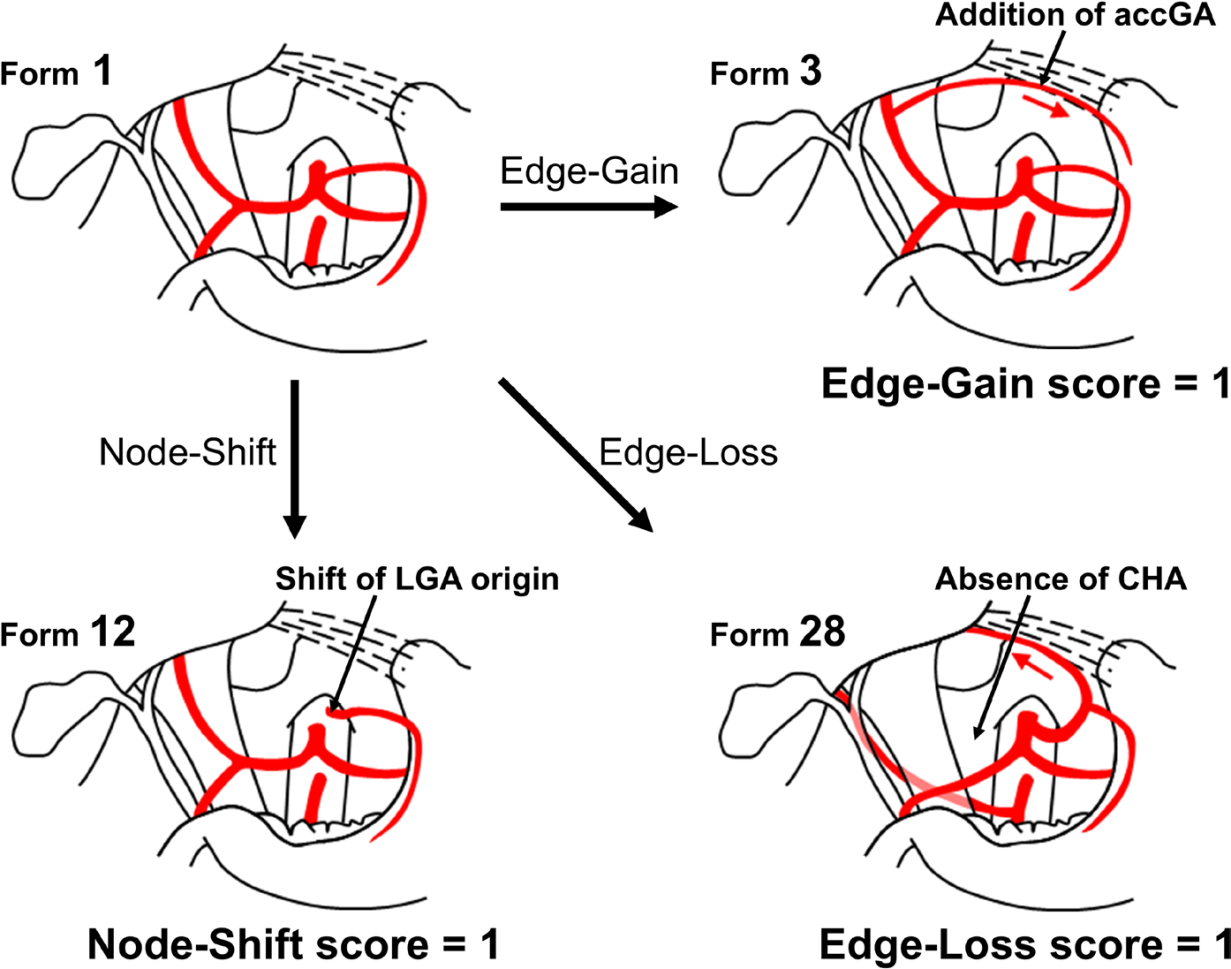
Definition of topological deviation parameters. Definition of the three parameters used to quantify topological deviation from the standard arterial configuration (Type 1 [Form 1]). Node‐Shift represents the displacement of a branching point relative to its position in the standard configuration. Edge‐Gain represents the presence of additional arterial branches, such as accessory arteries. Edge‐Loss represents the absence of the arterial branches that are normally present in the standard configuration. Each illustrated example demonstrates a single‐step change and yields a score of one for the corresponding parameter.

The three parameters used in this study were defined as follows (Figure 3):

- Node‐Shift Score: The number of steps in which the position of a branching node is shifted relative to its position in the standard configuration. This reflects the changes in the relative positional relationships among nodes. A “step” was defined as the displacement of a branching node along the predefined arterial branching graph. The step count corresponds to the shortest path distance between the original node position in the standard configuration and the displaced node position in a given branching form.
- Edge‐Gain Score: The number of additional branches, such as accessory arteries, that are absent in the standard configuration. An Edge‐Gain was recorded whenever a branch appeared in a given subtype but was not present in the standard configuration.
- Edge‐Loss Score: The number of arterial branches that are normally present in the standard configuration but are absent in a given subtype. An Edge‐Loss was recorded whenever a branch present in the standard configuration was absent in the subtype being evaluated.

By combining these three indices, we aimed to comprehensively evaluate the extent to which each branching pattern deviated topologically from the standard configuration. Worked examples illustrating how the Node‐Shift, Edge‐Gain, and Edge‐Loss Scores were calculated for each branching form are provided in Figure S1.

In this framework, replaced hepatic arteries (e.g., the right hepatic artery or left hepatic artery arising from the SMA or LGA) were treated as topologically equivalent to accessory hepatic arteries and were therefore counted as Edge‐Gain events. When such configurations were accompanied by the absence of the CHA from the celiac trunk, this absence was counted as an Edge‐Loss event.

### 2.4 Statistical Analysis

First, the three topological parameters (Node‐Shift, Edge‐Gain, and Edge‐Loss) were calculated for all 28 subtypes of the celiac trunk–SMA system described by Adachi [1] by using the standard configuration as the reference. Each parameter quantitatively represents the degree of topological deviation of a given subtype from the standard pattern.

Thereafter, the relationships between these parameters and reported occurrence frequencies [1] were evaluated using a two‐step regression analysis. In the first step, subtypes with Edge‐Loss = 0 (Forms 1–23) were analyzed by using Node‐Shift and Edge‐Gain as explanatory variables and occurrence frequency as the dependent variable. This subset represented relatively basic topological variations without branch loss and was analyzed to examine how the frequency decreased under conditions of branch preservation. In the second step, all 28 subtypes were analyzed by adding Edge‐Loss as a third explanatory variable to assess the frequency changes across a broader range of topological variations.

Linear, exponential, and logarithmic regression models were applied to account for the possibility of nonlinear relationships between the topological parameters and frequency. All analyses were performed using Python (version 3.11), and model fit was evaluated using the coefficient of determination (*R* ^2^).

### 2.5 Ethics Statement

Institutional Review Board approval was not required because this study used previously published, aggregated data and excluded human subjects or other identifiable personal information.

## 3 Results

### 3.1 Topological Scoring of the Branching Patterns

**FIGURE 4:**
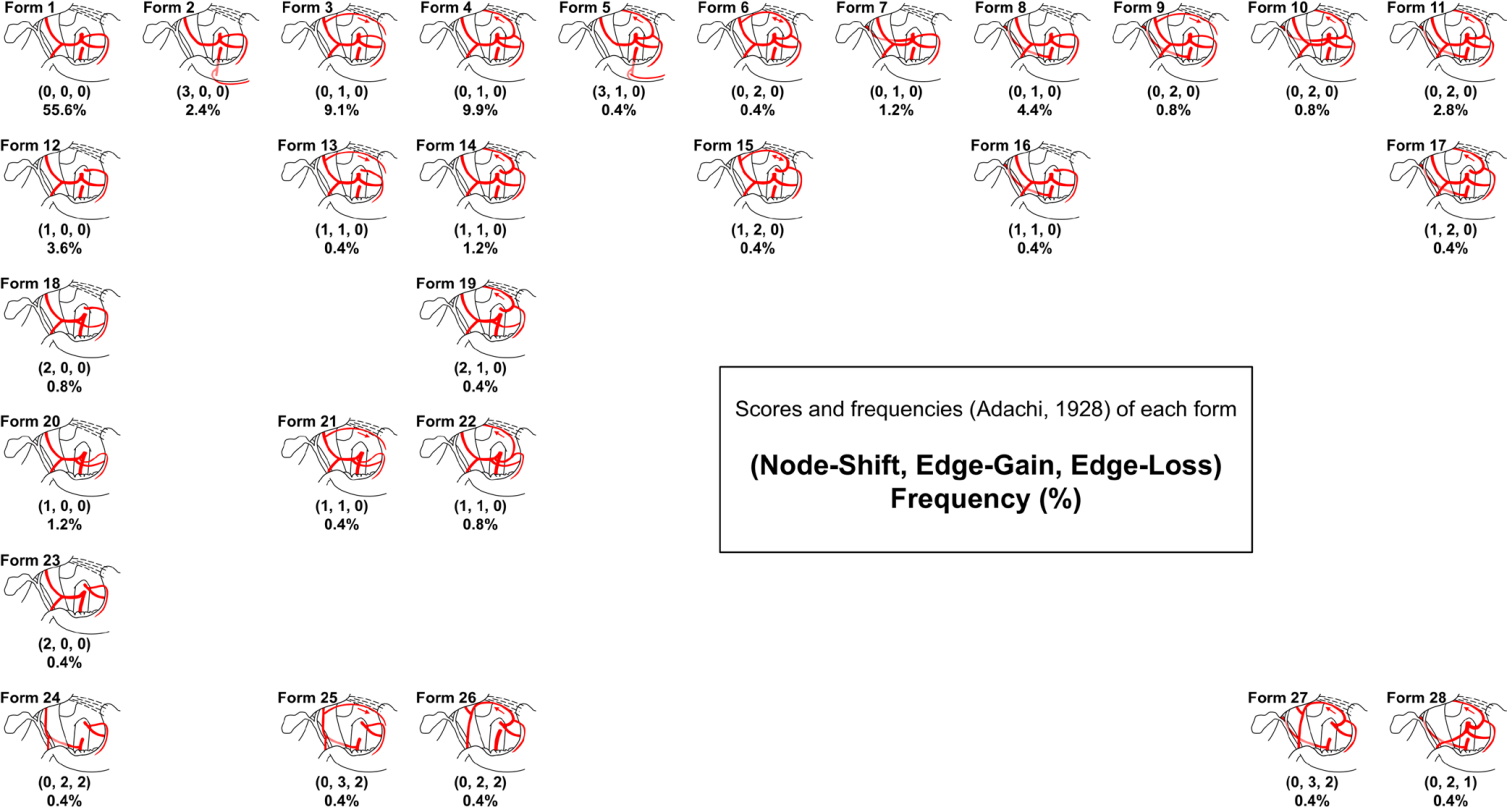
Topological scores and frequencies of the Adachi subtypes. Schematic representation of all 28 Adachi subtypes of the celiac trunk–superior mesenteric artery (SMA) system and their corresponding topological scores and reported occurrence frequencies [1]. For each form, the triplet indicates the scores for (Node‐Shift, Edge‐Gain, Edge‐Loss), followed by frequency (%). This summary illustrates that increasing the topological deviation from the standard configuration is associated with progressively lower occurrence frequencies.

Table 1 shows the results of the topological scoring using Node‐Shift, Edge‐Gain, and Edge‐Loss for all 28 subtypes of the celiac trunk–SMA system, together with their occurrence frequencies. Figure 4 presents the schematic diagrams of the vascular configurations of each subtype, along with the corresponding topological scores and frequencies.

**TABLE 1:** Topological edit distance and frequency of anatomical variations in the celiac trunk–superior mesenteric artery system.

Form	Node‐Shift	Edge‐Gain	Edge‐Loss	Frequency Adachi [1] (%)
1	0	0	0	55.6
2	3	0	0	2.4
3	0	1	0	9.1
4	0	1	0	9.9
5	3	1	0	0.4
6	0	2	0	0.4
7	0	1	0	1.2
8	0	1	0	4.4
9	0	2	0	0.8
10	0	2	0	0.8
11	0	2	0	2.8
12	1	0	0	3.6
13	1	1	0	0.4
14	1	1	0	1.2
15	1	2	0	0.4
16	1	1	0	0.4
17	1	2	0	0.4
18	2	0	0	0.8
19	2	1	0	0.4
20	1	0	0	1.2
21	1	1	0	0.4
22	1	1	0	0.8
23	2	0	0	0.4
24	0	2	2	0.4
25	0	3	2	0.4
26	0	2	2	0.4
27	0	3	2	0.4
28	0	2	1	0.4

**FIGURE 5:**
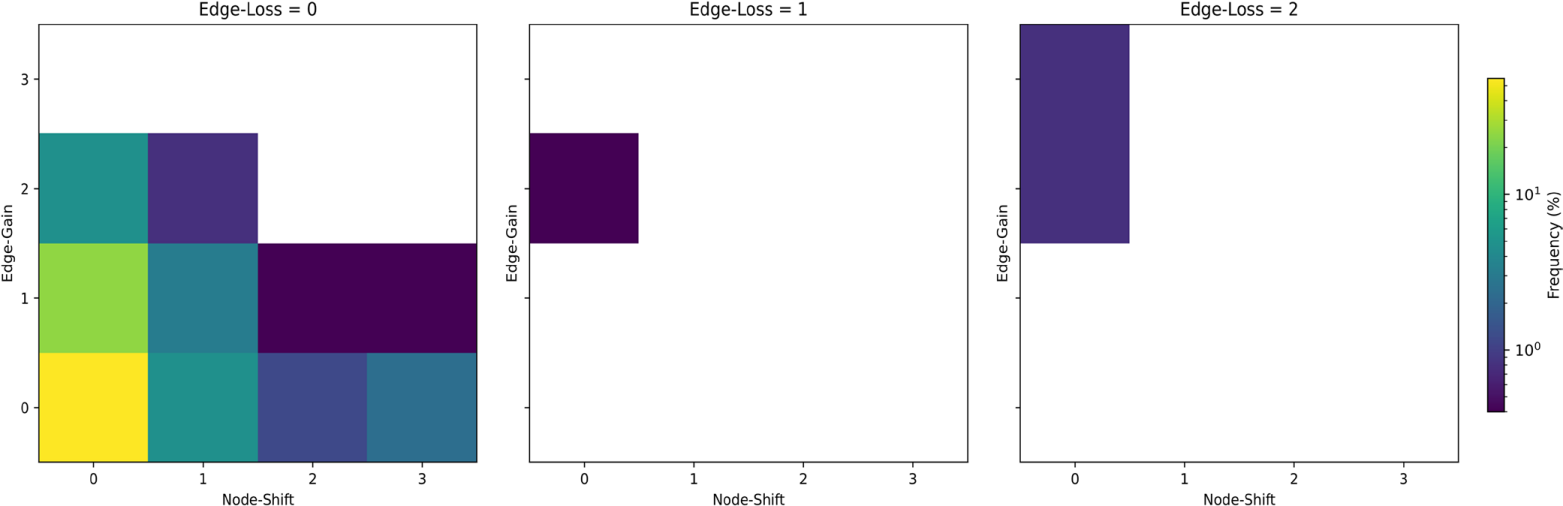
Distribution of occurrence frequency across topological parameter space. Heat maps illustrating the relationship between topological parameters and occurrence frequency. The Node‐Shift and Edge‐Gain are plotted on the horizontal and vertical axes, respectively, and the frequency (%) is indicated by color intensity. Separate panels show Edge‐Loss Scores of = 0, 1, and 2. High‐frequency patterns are concentrated near the standard configuration, whereas patterns with greater topological deviation are rare.

To visualize the relationships between the three topological parameters and the occurrence frequency, the parameter values were plotted along the axes, and the frequency was displayed as a color map (Figure 5). As shown in the figure, the occurrence frequency decreased consistently as the magnitude of the topological deviation from the standard configuration increased. Therefore, higher Node‐Shift Scores and increasingly complex combinations of Edge‐Gain and Edge‐Loss were associated with lower frequencies.

Within the range of Edge‐Loss = 0, a relatively wide variety of branching patterns was observed; however, subtypes with a Node‐Shift Score of 0 or 1 accounted for the majority of high‐frequency patterns, whereas frequencies declined markedly when Node‐Shift ≥ 2. Subtypes with an Edge‐Loss Score of one or two were all clustered at very low frequencies, indicating that topological changes involving branch loss rarely occur in this arterial system.

### 3.2 Regression Analysis: Subtypes 1–23 (Edge‐Loss = 0)

We first examined the relationship between Node‐Shift, Edge‐Gain, and occurrence frequency in subtypes 1–23, which do not involve branch loss. In the linear regression and log‐linear models, the coefficients of determination were 0.331 and 0.526, respectively. Although both Node‐Shift and Edge‐Gain showed significant negative associations with frequency in these models (both *p* < 0.05), the overall goodness of fit was limited.

**FIGURE 6:**
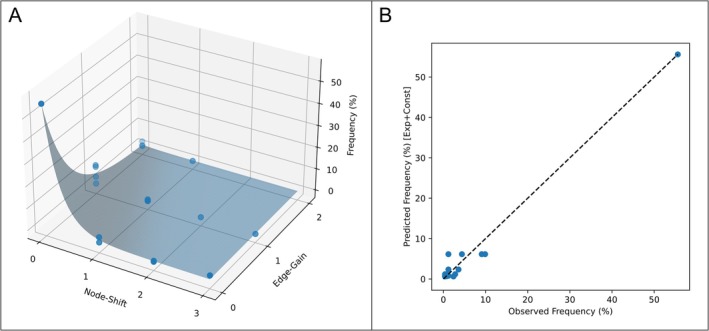
Exponential relationship between topological deviation and occurrence frequency. (A) Three‐dimensional plot showing the observed occurrence frequencies of branching patterns without branch loss (Edge‐Loss = 0; Forms 1–23) as a function of Node‐Shift and Edge‐Gain, together with the fitted exponential regression surface. (B) Comparison between observed and predicted frequencies derived from the exponential regression model with a constant term. The dashed line represents the line of identity. The close correspondence indicates that even modest increases in topological deviation from the standard arterial configuration are associated with a rapid decline in occurrence frequency.

By contrast, the exponential model with a constant term (Exponential + Constant) remarkably captured the frequency decline associated with increasing Node‐Shift and Edge‐Gain and yielded an *R* ^2^ of 0.979, the highest among the tested models (Figure 6A). The estimated model is expressed as follows:

$$\text{Freq}=54.97\cdot e^{\left(-3.463\cdot \text{NodeShift}-2.299\cdot \text{EdgeGain}\right)}+0.630.$$

A comparison between the predicted and observed frequencies demonstrated an almost perfect 1:1 correspondence (Figure 6B), quantitatively indicating that even modest increases in the topological deviation from the standard configuration resulted in a rapid decline in occurrence frequency.

These findings indicate that among subtypes without branch loss, the topological complexity defined by the combination of Node‐Shift and Edge‐Gain appears to be a necessary component for explaining the observed exponential trend in occurrence frequency. However, it is unlikely to represent a sufficient condition on its own.

### 3.3 Regression Analysis: All 28 Subtypes

We then extended the analysis to all 28 subtypes, including those with Edge‐Loss. In the linear regression and log‐linear models, the coefficients of determination were 0.319 and 0.537, respectively. In both models, Node‐Shift and Edge‐Gain retained significant negative effects on the frequency; however, the overall model fit remained limited. Edge‐Loss was not a statistically significant explanatory variable in either model, thus indicating that branch loss represents an extremely rare type of topological change. Therefore, branch loss contributes little to explaining the frequency variability.

By contrast, the exponential model (Exponential + Constant) showed excellent performance, maintaining a high *R* ^2^ of 0.979, which was comparable to that observed in the analysis of subtypes 1–23. The estimated model for all 28 subtypes is expressed as follows:

$$\text{Freq}=55.034\cdot e^{\left(-3.463\cdot \text{NodeShift}-2.286\cdot \text{EdgeGain}-21.302\cdot \text{EdgeLoss}\right)}+0.565.$$

In this formulation, the large negative coefficient associated with Edge‐Loss reflects the mathematical treatment whereby subtypes involving branch loss, which rarely occur, are collectively shifted toward extremely low frequencies. More importantly, the dominant gradient of the frequency distribution was already established by the combined effects of the Node‐Shift and Edge‐Gain, as observed in the analysis of subtypes 1–23.

Branching variations in the celiac trunk–SMA system are consistently governed by an exponential relationship between the occurrence frequency and topological deviation and are primarily driven by branch addition (Edge‐Gain) and shifts in branching position (Node‐Shift). This trend holds across all subtypes regardless of the presence or absence of branch loss, thus suggesting that a robust and generalizable principle underlies the formation of arterial branching variations.

## 4 Discussion

We demonstrated that the diverse branching variations observed in the celiac trunk–SMA system can be systematically explained by their topological distance from the standard configuration. Specifically, we showed that the magnitude of topological change, which was defined by Node‐Shift, Edge‐Gain, and Edge‐Loss, is associated with an exponential decline in occurrence frequency, with an exceptionally high coefficient of determination (*R* ^2^ = 0.979). Importantly, the scoring framework introduced in this study provides a quantitative representation of topological deviation from the standard arterial configuration. This approach allows branching patterns that have traditionally been described qualitatively within classification systems to be compared systematically along a continuous scale of structural modification. These findings indicate that the intrinsic topological complexity of a branching pattern is a major factor underlying its likelihood of occurrence. Branching variations that can be obtained from the standard pattern through minimal structural modifications are relatively common, whereas configurations requiring multiple shifts in the branching position and/or the addition or loss of branches rapidly are rare. Therefore, the diversity of celiac trunk–SMA branching patterns cannot be adequately explained by a purely random model and instead follows a reproducible probabilistic trend that can be described by a unified mathematical function.

The branching variations of the celiac trunk and SMA have classically been organized using the six types (28 forms) proposed by Adachi [1], and hepatic arterial anatomy has further been systematized using the widely adopted classifications of Michels (1966) and Hiatt (1994) [1, 2, 10]. These classification systems continue to serve as the foundation for understanding abdominal arterial anatomy. Recent anatomical and radiological studies based on cadaveric dissection or computed tomography (CT) have further refined the catalog of branching patterns and their frequencies [3, 4, 5, 14, 15, 16, 17]. Although these studies have been essential for documenting the spectrum and occurrence frequency of anatomical variants, they have generally focused on descriptive classifications. As a result, the fundamental question of why certain branching patterns are common, whereas others are exceedingly rare, remains largely unanswered.

Attempts to quantify arterial variation have predominantly relied on geometric parameters, such as vessel length, diameter, and interorigin distances [12, 18]. Although geometric morphometric approaches are powerful tools in anatomical research [19, 20], particularly for the analysis of organ and skeletal morphology, these approaches are limited in their ability to capture the essential features of arterial branching, namely, the connectivity structure itself (i.e., which branches arise from which parent vessels). Connectivity is an inherent topological property in arterial systems. By introducing Node‐Shift, Edge‐Gain, and Edge‐Loss as explicit topological descriptors, this study provides a framework that directly quantifies structural changes in branching architectures. The observed exponential relationship between topological complexity and frequency suggests that branching variations may be influenced by the cumulative “cost” associated with the formation process of developmental patterns, including branch addition, loss, or positional change, rather than arising solely from random geometric fluctuation. This approach allows branching patterns to be understood not as an unordered list of variation but as elements distributed along a continuous topological spectrum governed by a common probabilistic rule. Importantly, the present framework does not assume equal biological weighting among Node‐Shift, Edge‐Gain, and Edge‐Loss. Instead, the regression model allows the relative contributions of these parameters to be estimated empirically from the observed distribution of branching patterns.

From a developmental perspective, branching variations of the celiac trunk and SMA have traditionally been interpreted using the hypothesis of Tandler [21]: multiple arterial roots arising from the dorsal aorta, together with longitudinal anastomoses, undergo selective persistence or regression to generate a variety of branching patterns [22, 23]. This concept has been valuable for demonstrating the theoretical possibilities of many configurations. Although the model of Tandler can account for a range of conceivable patterns, it does not explain the marked skew in the observed frequencies; in other words, it describes what can form but not what tends to form. In this sense, Tandler's hypothesis provides a morphogenetic explanation of how different configurations may arise, whereas the present framework addresses the statistical distribution of those configurations by quantifying their topological deviation from the standard pattern.

In parallel, topological analyses of vascular networks have emerged in recent years, particularly in studies of microvascular systems, where connectivity patterns are treated using mathematical graph theory [24, 25, 26]. However, these analyses have primarily focused on microcirculatory networks and have not been applied to macro‐level arterial branching patterns, such as those of the celiac trunk–SMA system. To the best of our knowledge, no study has systematically quantified the major arterial branching variations by using explicit topological distance measures. The three parameters introduced in the current study provide such a framework, and the strong exponential relationship indicates that increasing topological deviation is consistently penalized by a rapidly declining occurrence frequency. The findings of this study parallel those of our previous work on aortic arch branching variations, in which a similar exponential relationship between topological distance and frequency was observed [11]. Importantly, the present analysis represents an independent application of the same topological framework to a different arterial system, thereby providing cross‐regional validation of the proposed probabilistic model of vascular branching variation. Therefore, a shared mathematical principle governs branching variations across different regions of the arterial system, from the thoracic vasculature to the abdominal vasculature.

In addition to anatomical and developmental implications, our findings are clinically relevant in the field of hepatobiliary–pancreatic surgery. Variations in the celiac trunk, SMA, and hepatic arteries directly influence the surgical strategy for hepatectomy, pancreatoduodenectomy, distal pancreatectomy, liver transplantation, and complex oncological resections. Accordingly, the precise preoperative recognition of vascular anatomy has been emphasized as a core component of “precision anatomy” in modern hepatobiliary surgery [27, 28]. Imaging‐based studies using CT angiography have demonstrated that arterial variations are common and that unrecognized variations may increase the risk of intraoperative vascular injury or postoperative ischemic complications [16, 29, 30].

This study provides a quantitative framework for the interpretation of anatomical variations. Rather than simply enumerating variant types, our model allows each branching pattern to be positioned along the scale of topological complexity. Therefore, extremely rare configurations are understood not as arbitrary anomalies but as structures that would require multiple steps during developmental pattern formation and are expected to have a markedly reduced occurrence frequency. This perspective may provide a conceptual basis for interpreting the extent to which a patient's anatomy deviates from the standard pattern. Moreover, because Node‐Shift, Edge‐Gain, and Edge‐Loss are not tied to any single classification system, the framework can be applied to previously unclassified or novel branching patterns. In the future, the automated extraction of branching topology from preoperative imaging followed by topological scoring may allow visualization of anatomical complexity and could potentially assist in identifying regions that require heightened attention during surgery. However, the clinical utility of this framework remains hypothetical and will require validation using clinical datasets.

This study has a few limitations. First, the analysis was restricted to the 28 subtypes defined in the classification of Adachi [1], and extremely rare variations outside this framework were not included. Second, the original dataset of Adachi [1] was primarily based on Japanese individuals, and population‐specific differences may not have been fully represented. Third, the finite sample size (*n* = 252) imposed a minimum frequency resolution of approximately 0.4%, which may have affected the apparent strength of the exponential fit among the rare subtypes.

In conclusion, we formalized the branching diversity of the celiac trunk–SMA system using three topological parameters (Node‐Shift, Edge‐Gain, and Edge‐Loss) and demonstrated that the occurrence frequency of arterial branching variations declines exponentially as topological complexity increases. These findings indicate that vascular branching patterns follow a reproducible probabilistic structure shaped by developmental and anatomical constraints rather than arising randomly. The topological framework proposed in this study provides a quantitative basis for interpreting arterial branching variation beyond traditional descriptive classifications and offers a foundation for future studies of vascular development and anatomical variation.

## Funding

The authors have nothing to report.

## Conflicts of Interest

The authors declare no conflicts of interest.

## Supporting information


**Figure S1:** Scoring examples for all 28 branching forms based on the proposed topological framework.
The standard configuration (Form 1) is used as the reference. Yellow curved arrows indicate node shifts relative to the standard configuration, and the number of steps corresponds to the shortest path distance along the arterial branching graph. Thick yellow edges represent gained branches (Edge‐Gain), whereas dashed gray segments represent lost branches (Edge‐Loss). The total Node‐Shift, Edge‐Gain, and Edge‐Loss values assigned to each form are shown below each diagram.

## Data Availability

The data that support the findings of this study are available from the corresponding author upon reasonable request.
